# Trompe l’oeil electrocardiogram

**DOI:** 10.1007/s12471-017-0952-9

**Published:** 2017-01-19

**Authors:** E. Ströker, C. de Asmundis, G. B. Chierchia, P. Brugada

**Affiliations:** Heart Rhythm Management Centre, University Hospital Brussels, Free University of Brussels, Brussels, Belgium

A 59-year-old male patient was referred to our centre for a second opinion concerning a recurrent (pre)syncopal state. Previous external cardiac, carotid and neurological non-invasive evaluation did not lead to any aetiology or further investigations. His previous medical history included propafenone intake (300 mg/day) for palpitations, documented as frequent premature atrial extrasystoles. He presented to our clinic after stopping propafenone two weeks earlier (and stated that he actually felt better without it). The baseline electrocardiogram (ECG) showed sinus rhythm, left QRS axis (−65°) and a wide QRS interval (135 ms) (Fig. 1).

**Fig. 1:**
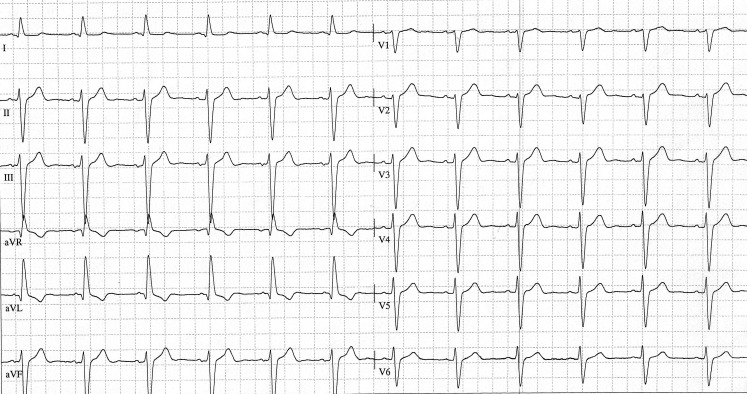
Baseline ECG showing a left anterior hemiblock pattern with a wide QRS interval

What would be your next step towards a correct diagnosis?

## Answer

You will find the answer elsewhere in this issue.

